# Supplementary figures and images for: Exact neural mass model for synaptic-based working memory
Source: PLoS Comput Biol. 2020 Dec 15;16(12):e1008533. doi: 10.1371/journal.pcbi.1008533 (PMC7771880; doi:10.1371/journal.pcbi.1008533)

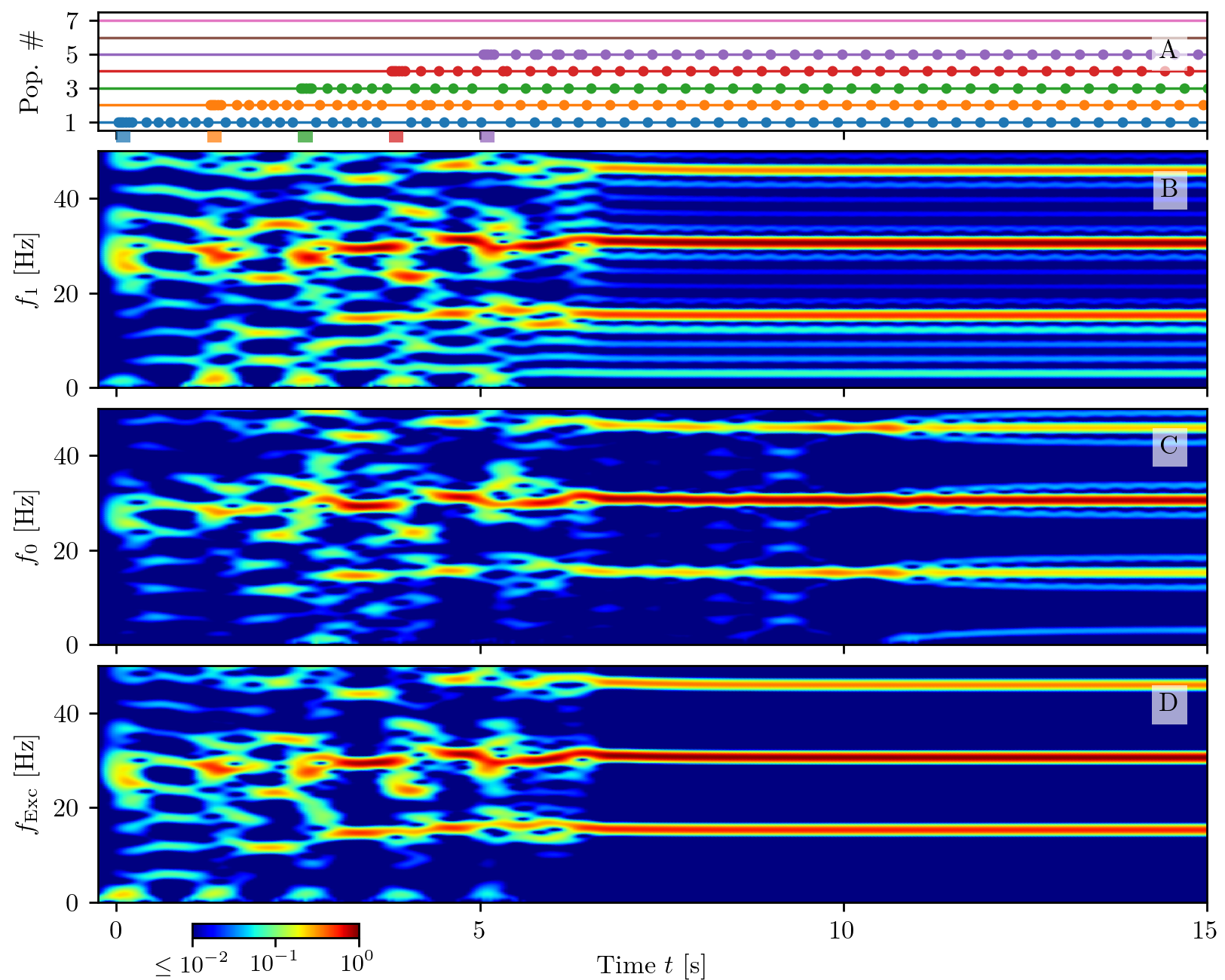

Supplement: S1 Fig — Response of the system when NL = 5 excitatory populations are subsequentely stimulated at a presentation rate of 0.8 Hz. Population bursts of excitatory populations (A): horizontal lines in absence of dots indicate quiescence phases at low firing rates rk for populations k = 1, …7. Dots mark PBs of the corresponding population. The coloured bars on the time axis mark the starting and ending time of stimulating pulses, targeting each population. Spectrograms of the mean membrane potential v1(t) (B), v0(t) (C) and of the mean membrane potentials averaged over all the excitatory populations (D); the corresponding frequencies have been denoted as f1, f0 and fExc. Parameter values as in Fig 8. (TIF) [file pcbi.1008533.s001.tif]
